# The diet of the first Europeans from Atapuerca

**DOI:** 10.1038/srep43319

**Published:** 2017-02-27

**Authors:** Alejandro Pérez-Pérez, Marina Lozano, Alejandro Romero, Laura M. Martínez, Jordi Galbany, Beatriz Pinilla, Ferran Estebaranz-Sánchez, José María Bermúdez de Castro, Eudald Carbonell, Juan Luís Arsuaga

**Affiliations:** 1 Departament de Biologia Evolutiva, Ecologia i Ciencies Ambientals, Secció Zoologia i Antropologia Biològica, Universitat de Barcelona. Av. Diagonal, 643, 08028 Barcelona, Spain; 2 Institut Català de Paleoecologia Humana i Evolució Social (IPHES), Zona Educacional 4 (Edifici W3), Campus Sescelades, 43007 Tarragona, Spain; 3 Àrea de Prehistòria, Universitat Rovira i Virgili, Av. Catalunya, 35, 43002 Tarragona, Spain; 4 Departamento de Biotecnología, Facultad de Ciencias, Universidad de Alicante, Ap. C. 99, 03080 Alicante, Spain; 5 Center for the Advanced Study of Human Paleobiology, Department of Anthropology, The George Washington University, 800 22nd Street NW, Ste 6000, Washington DC 20052, USA; 6 Centro Nacional de Investigación sobre Evolución Humana (CENIEH), Paseo Sierra de Atapuerca, 09002 Burgos, Spain; 7 UCL Anthropology, 14 Taviton Street, London, WC1H 0BW, UK; 8 Centro Mixto UCM-ISCIII de Evolución y Comportamiento Humanos, Universidad Complutense de Madrid–Instituto de Salud Carlos III, 28029 Madrid, Spain; 9 Departamento de Paleontología, Facultad de Ciencias Geológicas, Universidad Complutense de Madrid, 28040 Madrid, Spain

## Abstract

Hominin dietary specialization is crucial to understanding the evolutionary changes of craniofacial biomechanics and the interaction of food processing methods’ effects on teeth. However, the diet-related dental wear processes of the earliest European hominins remain unknown because most of the academic attention has focused on Neandertals. Non-occlusal dental microwear provides direct evidence of the effect of chewed food particles on tooth enamel surfaces and reflects dietary signals over time. Here, we report for the first time the direct effect of dietary abrasiveness as evidenced by the buccal microwear patterns on the teeth of the Sima del Elefante-TE9 and Gran Dolina-TD6 Atapuerca hominins (1.2–0.8 million years ago − Myr) as compared with other Lower and Middle Pleistocene populations. A unique buccal microwear pattern that is found in *Homo antecessor* (0.96–0.8 Myr), a well-known cannibal species, indicates dietary practices that are consistent with the consumption of hard and brittle foods. Our findings confirm that the oldest European inhabitants ingested more mechanically-demanding diets than later populations because they were confronted with harsh, fluctuating environmental conditions. Furthermore, the influence of grit-laden food suggests that a high-quality meat diet from butchering processes could have fueled evolutionary changes in brain size.

The dietary strategies of the European Lower Pleistocene hominins from Atapuerca (Burgos, Spain) have been inferred solely from faunal assemblages that were found in the same levels where the human remains were unearthed. The TE9 Unit at the Sima del Elefante site (1.2 Myr) has yielded a broad range of medium and large-sized mammals and even tortoises that show anthropogenic modifications, which indicate that they were part of the hominin diet^1^,^2^. Moreover, the TD6 level at the Gran Dolina site (0.8–0.96 Myr) has provided evidence not only of hunted and scavenged mammals^3^ but also of butchered and eaten human remains, which is the most ancient evidence of human cannibalism^4^. However, the feeding habits of these hominins have not been inferred from a wide range of sources of information similar to the Middle Pleistocene European populations. Dental wear^5^,^6^ and isotopic signals^7^ have provided valuable information of Middle Pleistocene species. The isotopic signals are indicative of mainly carnivorous dietary regimens for Neandertal populations. However, carnivorous regimens contrast with the buccal dental microwear analyses that show dietary habits that include significant amounts of abrasive plant foods that cause highly abrasive loads on enamel surfaces compared with modern hunter-gatherer populations^8^,^9^. Because of these contradictory interpretations and because most of the academic attention^5^ has focused on Neandertals, an independent and comprehensive reconstruction of dietary ecology in Lower Paleolithic European hominins is necessary. We therefore apply a novel approach that is based on buccal dental microwear to reconstruct the dietary habits of the well-documented first inhabitants of Europe and compare them with other Pleistocene species.

Buccal dental microwear refers to the microscopic-scale (10^−6^ m) wear on non-working enamel surfaces of premolar and molar teeth that is caused by dietary abrasions^10^,^11^,^12^. Scratches of different lengths and orientations are formed across buccal enamel surfaces during food breakdown by particles such as silica phytoliths or exogenous quartz grits^11^,^13^. The type and amount of abrasives in chewed foods have been shown critical to buccal enamel scratch formation processes^11^,^12^, which demonstrate a relationship between buccal microwear patterns and abrasive properties despite the lack of attritional tooth-to-tooth contact^9^,^11^. The predicted critical loads required to fracture enamel in humans exceed 500 Newtons (N), whereas the loads required to produce individual microwear traces are in the order of milli-Newtons (mN)^10^. In addition, scratch formation is aided by particle kinetic energy^10^, producing sufficient load for abrasives to scratch buccal enamel surfaces. Furthermore, *in vivo* models have shown that microwear features are produced on buccal enamel surfaces of volunteers consuming soft diets and that scratch formation is a dynamic process that develops over time and reflects long-term trends in dietary habits^11^. The scratch densities on buccal enamel surfaces clearly relate to the increased abrasiveness of the dietary habits of hunter-gatherer and farmer populations^9^,^12^.

The hominins from Sima del Elefante (ATE9−1; *n* = 1) and Gran Dolina-TD6 (*H. antecessor; n* = 7) are both from the Sierra de Atapuerca (Burgos, Spain) and cover a chronological range between 1.2 and 0.8 Myr^14^. The buccal microwear patterns from premolar and molar teeth of these specimens were compared with the buccal microwear patterns of Lower and Middle Pleistocene hominins (Table 1 and Supplementary Text 1), including the fossil specimens of *H. ergaster* (1.5–0.7 Myr; *n* = 7), *H. heidelbergensis* (0.6–0.2 Myr; *n* = 21, which comprise individuals from Arago, Kabwe, Pontnewydd and Sima de los Huesos), and *H. neanderthalensis* from the Iberian Peninsula (0.05–0.03 Myr; *n* = 6, including Cova Foradà, El Sidrón, Figueira Brava and Sima de las Palomas). Scanning Electron Microscopy (SEM) micrographs of well-preserved buccal enamel surfaces were recorded at 100× magnification. Only buccal enamel surfaces that clearly showed *ante-mortem* scratches without sedimentary trampling or chemical alterations^9^,^11^,^12^ were considered for microwear analyses (Fig. 1a). Diet-induced buccal microwear shows a scratched pattern with scratches of various lengths and predominant occlusal-to-cervical orientation^11^. Instead, non-dietary post-depositional processes are readily identifiable, showing enamel cracks, abrasion effects from sedimentary particles with scratches usually larger in width (>20 μm), erosive effects erasing dietary-related microwear signatures, and enamel prism exposure^9^,^15^,^16^ (see Supplementary Text 2 and Fig. S1 for a detailed description on enamel preservation).

Buccal dental microwear was analyzed from selected SEM micrographs of each individual by using feature-based established protocols^8^,^9^. The buccal microwear patterns were defined by the density (NT), average length (XT, in μm), and standard deviation (σ) of the length (ST) of the overall scratches, as well as classified by 45° orientation intervals into vertical (V), horizontal (H), mesio-occlusal to disto-cervical (MD), and disto-occlusal to mesio-cervical (DM) scratch categories in density, length and their dispersion (σ) to identify the influence of dietary abrasiveness and inter-specific variability in microwear signatures^9^,^12^ (see Methods for a detail description).

## Results

**Figure 1:**
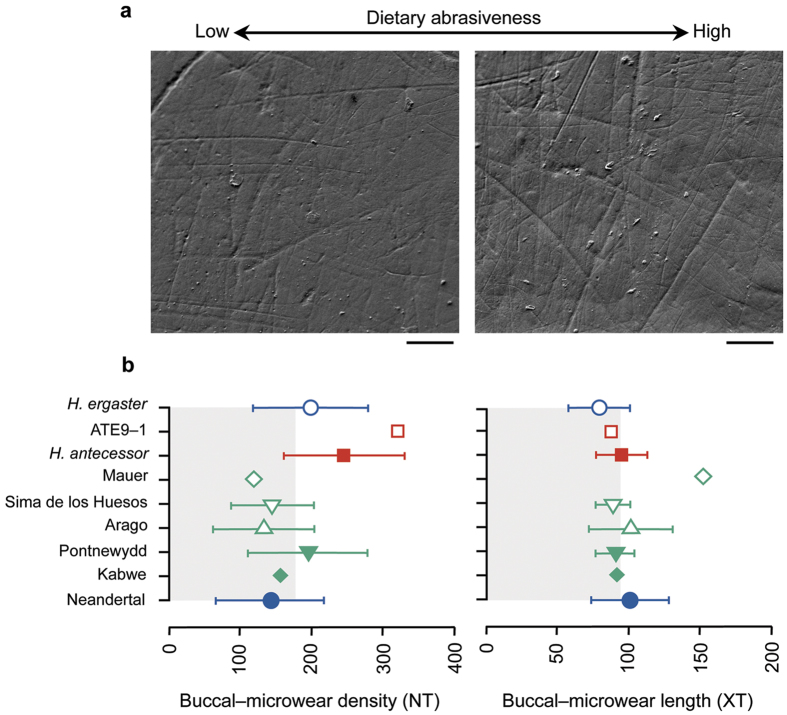
Buccal microwear pattern variability of the studied fossil groups. (**a**) The well-preserved buccal enamel surface of the El Sidrón Neandertal (the left SEM image) and *H. antecessor* (ATD6-5) specimens. Note the highly abraded surfaces in the *H. antecessor* H1 specimen. Each micrograph represents an enamel patch of 0.56 mm^2^ on the mandibular first molars at 100× magnification. The scale bar is 100 μm (common to both images). (**b**) Dot-plot showing the scratch density (NT) and average scratch length (XT, in μm) values for the analyzed species or individual. Interspecific differences in abrasiveness that relate to dietary habits are observed. The error bars denote ±1 standard deviation. The grey square areas delimit the mean values for all samples. See Table 1 and Supplementary Text 1 for the sample’s composition and details.

**Figure 2:**
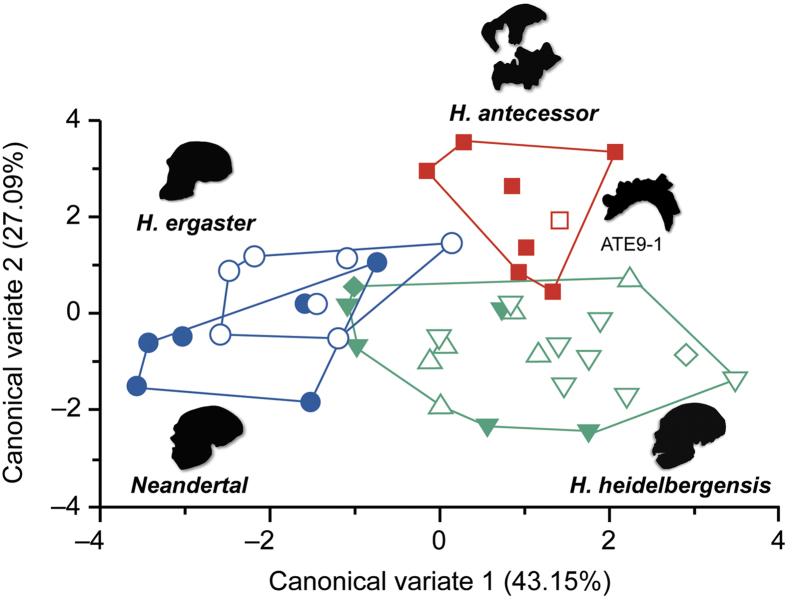
Bivariate plot of scores for the first two canonical variates (70.24% of total variance) showing interspecific variability in buccal microwear patterns. Note that *H. antecessor* individuals exhibit a distinct buccal microwear pattern that is characterized by high scratch densities, whereas *H. ergaster* more closely resembles Iberian Neandertals. Significant overlap is recorded among the Sima de los Huesos (open triangles pointing downwards), Arago (open triangles pointing upwards), and Pontnewydd (filled triangles) populations, which reflect similar dietary abrasiveness. The symbols are similar to the previous plots (see Fig. 1). All analyzed individuals are plotted. The isolated ATE9−1 (open square), Kabwe (BH1, filled diamond) and Mauer (Ma, open diamond) tooth specimens were classified *post-hoc* with the derived canonical variates. Convex hulls show the distribution limits of each considered species. See Table 1 and Supplementary Text 1 for a sample description.

We found significant variation in the overall multivariate model (MANOVA: Wilk’s λ = 0.108; *P* = 0.005) indicating that buccal microwear patterns vary among the species examined. The buccal microwear of the earliest hominins from Atapuerca stands out among other Pleistocene hominins for the higher total scratch density (NT = 254.13 ± 25.98; ± σ) compared with the samples of *H. ergaster* (NT = 198.43 ± 81.19), *H. heidelbergensis* (NT = 151.71 ± 67.16) and Iberian Neandertals (NT = 141.50 ± 75.61) (Fig. 1b) (extended data Tables S1–S3). Univariate analyses of variance (ANOVAs) comparing the group means revealed significant differences among the four species for the total (NT: *F* = 4.430, *P* = 0.009), horizontal (NH: *F* = 2.913, *P* = 0.047) and disto-mesial (NDM: *F* = 8.592, *P* = 0.0005) scratch densities. Pairwise comparisons (Tukey’s HSD *post-hoc* test) showed that *H. antecessor* differed from *H. ergaster* for NDM (*P* = 0.012), from *H. heidelbergensis* for NDM (*P* = 0.001) and NT (*P* = 0.009), and from Iberian Neandertals for NDM (*P* = 0.000) and NT (*P* = 0.035). The oldest species (1.5–0.8 Myr), including *H. ergaster*, ATE9–1 and TD6 hominins (*H. antecessor*), showed the highest densities of scratches, whereas *H. heidelbergensis* and Neandertals presented lower scratch density values on the buccal enamel surfaces. No significant differences were observed in the *H. heidelbergensis* populations (Arago, Pontnewydd and Sima de los Huesos specimens). A Canonical Variate Analysis (CVA) was conducted to maximizes the among species variation using a linear combination of microwear variables^9^. The CVA included 39 fossil specimens (excluding the specimens from ATE9–1, Kabwe and Mauer that were classified *post-hoc*), and all 15 microwear variables analyzed. The taxa compared, showing similar sample sizes (from 6 to 8 specimens), correspond to *H. ergaster*, the well-defined populations of *H. antecessor* and *H. heidelbergensis*, and the Iberian Neandertals. Five canonical variates were extracted (Wilks λ = 1.467, *P* = 0.039) (extended data Table S4). The first two CVs (Fig. 2) explained 70.247% of the total variance. Individual ANOVAs showed significant differences among the groups for both CV1 (43.153%; *F* = 19.951, *P* < 0.001) and CV2 (27.094%; *F* = 18.557, *P* < 0.001). CV1 mainly correlated (Pearson *r, P* < 0.05) with XDM (*r* = 0.392) and NMD (*r* = 0.330). The Gran Dolina-TD6 hominins formed a cluster with the highest CV1 values and were clearly distant from the *H. ergaster* and Iberian Neandertal specimens, which showed less and longer mesio-distal scratches (NMD and XMD, respectively). Otherwise, CV2 significantly correlated (Pearson *r, P* < 0.05) with NDM (*r* = 0.680) and NT (*r* = 0.553). All the density variables positively correlated with CV2, and most length variables (except XV) negatively correlated with this factor. Significant differences in CV2 scores were found between the earliest Gran Dolina specimens and all other groups because of overall higher density and shorter scratches. Furthermore, differences were found between *H. ergaster* and *H. heidelbergensis (P* = 0.016) but not with the Iberian Neandertals analyzed (*P* > 0.05). The *post-hoc* probability of correct classification of the CVA was 74.36% (33.33% after Jackknife cross-validation) (extended data Table S5). The Atapuerca ATE9−1 specimen was classified into the *H. antecessor* group with an 88.4% *post-hoc* probability, and Kabwe was grouped with *H. ergaster* with a probability of 95.8%. The Mauer individual was classified with varying probabilities as Sima de los Huesos (44.1%), Arago (38.6%) or Pontnewydd (15.0%) but not as *H. antecessor* (2.4%), *H. ergaster* or Neandertal (0% in both cases).

## Discussion

The clearly distinct position of the earliest hominins from Atapuerca supports the hypothesis that their dietary habits included higher amounts of fracture-resistant foods. Between 1.2 and 0.8 Myr ago, the paleoenvironmental records of Sierra de Atapuerca indicate a rich and stable ecosystem. Woodland areas with conifers and mesic Mediterranean trees dominated the region, and the hominins lived in open landscapes with an abundance of humid meadows and woodlands^17^. Cut and percussion marks in the long bones of mammals are evidence of the TE9 hominins´ ability to chew meat and bone marrow^18^. The wide spectrum of animals that were consumed has been interpreted as an opportunistic subsistence-behavior^1^. The mammal species diversity at Gran Dolina-TD6 (0.96–0.8 Myr), including herbivores, carnivores and cannibalized hominins, along with evidence of skinning, defleshing, marrow extraction and bone chewing activities suggest that hominin subsistence strategies included systematic hunting and corpse exploitation with a more developed Mode 1 technology than TE9^3^,^19^,^20^. In addition, recent evidence of *Celtis* seeds at this site indicates the consumption of wild plant matter^21^.

Buccal dental microwear patterns cannot discriminate the diet composition among populations, but it is clearly indicative of overall dietary-related habits, abrasive particle content (both intrinsic to foodstuffs and gritty contaminants) and food processing techniques, such as lithic technology or other food processing methods^11^,^12^,^22^. Current archaeological evidence indicates no regular use of fire for cooking in the European Middle Pleistocene until approximately 300,000–400,000 years ago^23^. Because of the absence of fire evidence at both Atapuerca sites, ATE9-1 and Gran Dolina-TD6 hominins have been assumed to have consumed food items raw^2^,^24^. This assumption is consistent with the presence of features on human and other animal bone surfaces that are caused by human chewing in Gran Dolina-TD6. This assumption is also consistent with microwear fabrics (pits and chipping) on the occlusal surfaces of the anterior teeth of ATE9-1 and *H. antecessor* that resulted from highly demanding dietary regimes with a heavy loading bite that is compatible with bone crushing to access the marrow^24^,^25^. Thus, non-thermal processed foods, including tough and/or hard items, as well as contaminant grit from the soil, are expected to have been part of the diets of *H. antecessor* more than *H. heidelbergensis*. Both *H. heidelbergensis* and Neandertals show a clear reduction in microwear densities compared with *H. antecessor* that may relate to the differences in food-processing techniques concerning the use of more advanced tool technologies (Modes 2 and 3 might be more efficient than Mode 1), which has already been suggested to interpret the interspecific microwear variability of the Sima de los Huesos specimens regarding Neandertals^8^,^9^. Accordingly, the distinct microwear patterns of *H. antecessor*, characterized by high microwear densities, suggest that it might have specialize in the consumption of harder and/or tougher foods (more mechanically-challenging) than *H. ergaster* and Neandertals. The microwear texture analyses on occlusal enamel surfaces of *H. ergaster* specimens have been suggested to be indicative of a broad-based diet with neither extremely hard nor tough foods^26^. The isotope-based dietary signal suggests that European Neandertals (~37,000–120,000 years ago) would have consumed significant amounts of animal proteins^7^. However, meat could not be an exclusive food item in Neandertal diets because the plant micro-remains that have been found in the dental calculus indicates that their diet would have included starch-rich plant foods, mainly from grass seeds and underground storage organs (USOs)^27^,^28^. The buccal microwear patterns of Neandertal teeth have been interpreted as indicative of the consumption of generalized diets that are consistent with the consumption of some plant foods^9^. A substantial preparation previous to ingestion may have resulted in a less-abrasive diet in Neandertals than in the *H. heidelbergensis* specimens, which place Neandertals in a range of buccal microwear signatures between archaic *H. heidelbergensis* hominins^8^,^9^ and modern human hunter-gatherer populations^12^.

Meat and USOs differ mechanically in resistance and fracture properties^22^, but their stiffness and abrasiveness during microwear formation depend greatly on food processing methods and bite forces^11^. The mechanical properties of abrasive particles in foodstuffs are responsible for enamel indentation^5^,^13^. Plant phytoliths seem less capable of fracturing enamel than quartz dust, which is directly involved in the overall scratching and erasing of enamel surfaces during long periods^11^,^29^. In this regard, the highly abraded buccal surfaces of *H. antecessor* could indicate the ingestion of a large amount of grit-laden foodstuffs.

The highly abraded enamel microwear patterns that have been observed in the earliest Atapuerca hominins (1.2–0.8 Myr ago) are clearly distinct from Iberian Neandertals and *H. heidelbergensis*. Our findings suggest that *Homo antecessor* could have specialized in the exploitation of tough, hard and brittle foodstuffs with adhered grit particles, which may include underground plants (including grit), collagen or connective tissue, and bone. This mechanically-demanding diet would have required strong shearing and grinding processes during food consumption, although unprocessed meat breakdown requires less chewing force than tough plant foods that increase the scratch formation rates^29^. Thus, hunting or scavenging to obtain animal resources^30^ may also be consistent with the highly abrasive microwear pattern that is observed. A high-quality diet including meat consumption may have not only fueled the energy gain that is needed to support an enlarged brain^31^, which is the case of *H. antecessor* with a brain size of approximately 1,000 cc compared with *H. ergaster* (764 cc)^32^, but also represented a significant food source in a highly demanding environment where preferred foods fluctuated seasonally. Furthermore, not-fully developed tool technologies for food preparation, either lithic or otherwise, may have resulted in scarcely processed foods that included high amounts of abrasives that would also contribute to the highly abraded enamel surfaces that were observed. Whatever the relative significance of each of these factors, the buccal microwear patterns that are shown by the earliest hominins from Atapuerca likely signal a highly demanding diet that evolved to cope with both ecological and cultural constraints.

## Methods

### Data acquisition

Original tooth crowns were cleaned with acetone and ethanol solutions using a cotton-ear-bud and air-dried prior to molding. Whole tooth crown molds were made with President Microsystem^TM^ polysiloxane vinyl impression material (Coltène-Whaledent Corp.) following standard procedures^33^,^34^. High-resolution casts were made with Epo-tek 301 (Epoxy Technologies, Inc. Billerica, MA) epoxy resin^34^. The casts were examined at 40× with a stereomicroscope to determine their suitability for microwear analysis. The casts were mounted on aluminum stubs and sputter-coated with a ~15 nm layer of gold-palladium and analyzed under Scanning Electron Microscopy (SEM) at 100× magnification, an 18–25 mm working distance (WD), and 15 kV of acceleration voltage^9^,^33^. Variations in the SEM WD did not affect the microwear feature measures because all analyzed images were cropped to exactly the same square area (see below). During scanning, the buccal enamel surface of each tooth crown was placed perpendicular to the electron beam, with the occlusal crown rim facing upwards in all SEM images. The micrographs were taken in the middle third of the crown to avoid both the occlusal and cervical thirds of the buccal crown surface^9^,^35^. The micrographs were cropped with Adobe Photoshop CS5 to exactly cover 0.56 mm^2^ (748.33 × 748.33 μm) of enamel surface, and all micrograph measurements were properly scaled prior to analysis^8^,^9^. A high-pass (50 pixels) filter and automatic grey level adjustment were applied to all cropped digital grey-scale selected micrographs to reduce shadows and enhance the image’s contrast^9^,^33^.

### Microwear analysis

Only micrographs showing well-preserved enamel surfaces, without *post-mortem* physical or chemical alterations, were selected for microwear analysis^9^,^15^,^16^ (Supplementary Text 2 and Fig. S1). Following previous procedures^8^,^9^,^12^, the length (in μm) and slope (with respect to the horizontal occlusal plane) of all observed scratches in the studied enamel patch were measured by using a semi-automated procedure with SigmaScan Pro 5.0 (SPSS^TM^) software, including scratches truncated by the edge of the micrographs^9^,^11^,^35^. Both parallel and overlapping scratches were measured as independent lineal abrasion units. Scratches that measured less than 10 μm in length (approximately 4 times the average width of the scratches) were discarded.

The buccal microwear variables measured included the scratch density (N), the average length (X) and the standard deviation of the length (S) of all the recorded scratches (T) in each analyzed micrograph (variables NT, XT and ST), as well as for the four 45° orientation categories (H, V, MD, DM)^8^,^35^. The classification of each scratch to an orientation category varied depending on the tooth position (upper, lower) and side (left, right) and on the *angle* of orientation with respect to the Cemento-Enamel Junction (CEJ, 0° of orientation)^35^ as follows: mesio-occlusal to disto-cervical (mesio-distal, MD) scratches (22.5° < *angle* ≤ 67.5° for upper, left or lower, right teeth; or 112.5° ≤ *angle* < 157° for upper, right or lower, left teeth); disto-occlusal to mesio-cervical (disto-mesial, DM) scratches (22.5° < *angle* ≤ 67.5° for upper, right or lower, left teeth; or 112.5° ≤ *angle* < 157° for upper, left or lower, right teeth); vertical (V) scratches (67.5° < *angle* < 11.5°) and, horizontal (H) scratches (157.5° ≤ *angle* ≤ 22.5°). Consequently, a total of 15 variables (NT, XT, ST, NV, XV, SV, NH, XH, SH, NMD, XMD, SMD, NDM, XDM, and SDM) accounted for the buccal-microwear pattern derived for each studied tooth.

Since the inter-observer error is a major concern in microwear research, both for the occlusal^36^ and buccal^37^ enamel surfaces, the micrographs of the specimens under study were measured by a single observer (LMM). However, inter-observer error analyses have not shown significant differences in the buccal microwear measurements among experienced researchers^37^.

### Statistical Analysis

All the studied variables passed the Kolmogorov-Smirnov normality tests (Z = 0.44−1.24; *P* > 0.05). A multivariate analysis of variance (MANOVA) was calculated with the values for every microwear density and length attributes as dependent variables, to determine the significance of the inter-specific variability in microwear signatures^9^,^11^. One-way analyses of variance (ANOVA) and *post hoc* pairwise comparisons that used the Tukey’s Honest Significant Difference (Tukey’s HSD) test were used in the ANOVA tests to check the inter-specific and population differences in the microwear patterns. Finally, a Canonical Variates Analysis (CVA) was performed to show the major trends in the buccal microwear patterns among the groups considered, not including the isolated teeth of the ATE9−1, Kabwe (BH1) and Mauer specimens for which the CV values were computed by using the functions that were derived from the analysis. The descriptive statistics and tests at α = 0.05 significance level were conducted by using Addinsoft XLSTAT-3.02.

## Additional Information

**How to cite this article:** Pérez-Pérez, A. *et al*. The diet of the first Europeans from Atapuerca. *Sci. Rep.* **7**, 43319; doi: 10.1038/srep43319 (2017).

**Publisher's note:** Springer Nature remains neutral with regard to jurisdictional claims in published maps and institutional affiliations.

## Supplementary Material

Suplementary Text 1 and 2

Supplementary Dataset 1

## Figures and Tables

**Table 1:** Fossil teeth samples included in the buccal microwear analysis.

Taxon and specimen	Known age (Myr)	Tooth
*Homo ergaster*	1.5–0.7	
KNM-ER 806		LM_1_
KNM-ER 807		RM^3^
KNM-ER 820		LM_1_
KNM-ER 992		RM_1_
KNM-WT 15000		RM_1_
OH 23		LP_4_
SK15		LM_1_
*Homo sp.*		
Atapuerca–Sima del Elefante (TE)	1.2	
ATE9-1		LP_4_
*Homo antecessor*		
Atapuerca–Gran Dolina (GD-TD6)	0.8	
H1		RM_1_ (ATD6-5)
H2		Lm^1^ (ATD6-14)
H3		LM^1^ (ATD6-69)
H5		RM_1_ (ATD6-94)
H7		LM_1_ (ATD6-96)
H10		LM_2_ (ATD6-113)
H11		Lm_1_ (ATD6-112)
*Homo heidelbergensis*	0.6–0.2	
Mauer		RM_1_
Atapuerca–Sima de los Huesos (SH)		
IV		RM^1^ (AT-3178)
VII		LM^2^ (AT-270)
VIII		LM^1^ (AT-3177)
XVII		RM^1^ (AT-20)
XIX		LM_1_ (AT-576)
XX		LM^1^ (AT-406)
XXII		RM^2^ (AT-588)
XXVI		RM_1_ (AT-561)
Arago		
A7		RP^4^
A13		RM_1_
A21		RM^2^
A40		RM_1_
A54		RM^1^
A69		RM_2_
Pontnewydd		
PN1		RM^2^
PN5		RP_4_
PN7		LP_3_
PN12		LM^1^
PN20		LP_3_
Kabwe		
BH1		RM^1^
*Homo neanderthalensis*	0.05–0.03	
Cova Foradà		
CF-1		LM^1^
El Sidrón		
SDR-007d		LM_1_
SDR-012		LM^1^
Sima de las Palomas		
SP-29		RM_2_
SP-59		LM_1_
Figueira Brava		
FB		LP^4^

A single premolar or molar tooth was used to represent each individual. The tooth samples comprised high-resolution casts that were made from the original specimens and were stored at the Zoology and Biological Anthropology section, Faculty of Biology (University of Barcelona). The specimen arrangement and dating results are from scientific publications (see Supplementary Text 1 for sample details).
