# Supplementary material for: The diet of the first Europeans from Atapuerca
Source: Sci Rep. 2017 Feb 27;7:43319. doi: 10.1038/srep43319 (PMC5327419; doi:10.1038/srep43319)
Supplement: Suplementary Text 1 and 2 [file srep43319-s1.doc]

**The diet of the first Europeans from Atapuerca**

**Authors:** Alejandro Pérez-Pérez^1*^, Marina Lozano^2,3*^, Alejandro Romero^4*^, Laura M. Martínez^1*^, Jordi Galbany^5^, Beatriz Pinilla^1^, Ferran Estebaranz^1^, José María Bermúdez de Castro^6,7^, Eudald Carbonell^2,8^, Juan Luís Arsuaga^8,9^

**Affiliations**

^1^Departament de Biologia Evolutiva, Ecologia i Ciencies Ambientals, Secció Zoologia i Antropologia Biològica, Universitat de Barcelona. Av. Diagonal, 643, 08028 Barcelona, Spain

^2^Institut Català de Paleoecologia Humana i Evolució Social (IPHES), c\ Marcelí Domingo s/n (Edifici W3), Campus Sescelades, 43007 Tarragona, Spain

^3^Àrea de Prehistòria, Dept. d’Història i d’Història de l’Art, Univ. Rovira i Virgili, Fac. de Lletres, Av. Catalunya, 35, 43002 Tarragona, Spain

^4^Departamento de Biotecnología, Facultad de Ciencias, Universidad de Alicante, Ap. C. 99, 03080 Alicante, Spain

^5^Center for the Advanced Study of Human Paleobiology, Department of Anthropology, The George Washington University, 800 22nd Street NW, Ste 6000, Washington DC 20052, USA

^6^Centro Nacional de Investigación sobre Evolución Humana (CENIEH), Paseo Sierra de Atapuerca, 09002 Burgos, Spain

^7^UCL Anthropology, 14 Taviton Street, London, WC1H 0BW, UK

^8^Centro Mixto UCM-ISCIII de Evolución y Comportamiento Humanos, Universidad Complutense de Madrid–Instituto de Salud Carlos III, 28029 Madrid, Spain

^9^ Departamento de Paleontología, Facultad de Ciencias Geológicas, Universidad

Complutense de Madrid, 28040 Madrid, Spain

**Supplementary text 1**

**Samples studied**

**Early *Homo* specimens from Africa**

The *Homo ergaster* taxon includes East and South African specimens that are dated from 1.89-0.90 million years (Myr) ago^1,2^. The dental sample for eastern Africa includes specimens from the Koobi Fora Formation (KNM-ER 806, KNM-ER 807, KNM-ER 820, KNM-ER 992) and the Noriokotome boy (KNM-WT 15000), as well as stratigraphic information and age estimates for hominins from the Turkana Basin^3–5^. In addition, the OH 23 specimen from the Olduvai Gorge was included, which is a partial mandible with a left P4-M2 that came from the Masek beds^6^, a layer above Bed IV, and it is estimated to be older than 0.78 Myr, perhaps 1.0-0.75 Myr^1,7^ old. Finally, the Swartkrans SK 15 specimen from Member 2 was placed in the *H. ergaster* taxon following several research studies^2,3,8^. The KNM-ER 807, KNM-ER 820, KNM-ER 992, KNM-WT 15000 and SK 15 specimens have been previously used for occlusal-microwear purposes^9^.

***Homo specimens* from Atapuerca**

The Sierra de Atapuerca, with a maximum altitude of 1,082 m above sea level, is located 14 km east of the city of Burgos (Spain). Several Pleistocene infillings can be found in this area, and two of these infillings are Trinchera del Elefante (TE) and Gran Dolina–TD6 (GD–TD6), which have provided the most ancient European human remains that are attributed to *Homo sp.* (1.2 Myr)^10,11^ and *H. antecessor* (0.9 Myr)^12^, respectively.

The TE site consists of a cave that is 18 m deep and up to 15 m wide, with a sedimentary sequence of 22 lithostratigraphic units that were mostly made by debris flow deposits^11^. A fragment of a mandible (ATE9–1) with a RI_2_ and L-RC_s_ that are preserved in place and an isolated lower left premolar (LP_4_) of the same individual were discovered in the TE9c level^11^. A hand phalanx and a small fragment of femur were recovered later from the same level^10^. Hominin fossils are associated with Mode 1 lithic technology, and faunal remains were dated from 1.2-1.1 Myr (MIS 37) ago based on paleomagnetism, cosmogenic nuclides and biostratigraphical data^11,13,14^.

The Gran Dolina (GD) cavity comprises 11 lithostratigraphic units, which are named from the oldest (TD1) to the youngest (TD11) levels^15^. The TD6 level has provided evidence of human remains^12^, which was recovered mixed with lithic artifacts^16^ and various macromammal taxa^17,18^. A descriptive and comparative study of the human dental remains that were recovered from GD–TD6 can be found in early reports^19–23^. Thermoluminiscence (TL) and Electron Spin Resonance (US-ESR) results provided a new date of 936,000 years ago (kyr) for TD7 (MIS 25). However, more recent dates suggest that the probable age of TD6 human fossils are from approximately 0.8-0.88 kyr ago during MIS 21, a warm and humid interglacial period, because they are located 0.1 m below the reported subchron^24,25^. At GD–TD6, approximately 170 human fossil remains are represented as belonging to 11 individuals (who range from 3 to 20 years old) who are assigned to the *H. antecessor* taxon of which the ATD6–5 mandible and teeth are the holotype^12,22,26^. *Homo antecessor* fossil specimens show a primitive morphological dental pattern that is comparable with the dental pattern of the early African *Homo* specimens that are dated between 1.8 and 1.4 Myr ago (including *H. habilis* and *H. rudolfensis*) as well as with *H. georgicus* (D211 and D2735) and the Sangiran mandibles^20^. The TD6 hominins seem to represent a European lineage that is distinct from other known African and Asian lineages, including the European Pleistocene Neandertal lineage, with some similarities to Chinese middle Pleistocene mandibles^20,22^.

***Homo heidelbergensis* specimens**

The *H. heidelbergensis* taxon was used here to refer to the European Middle Pleistocene populations, including hominins from Sima de los Huesos^27^, Arago^28,29^ and Pontnewyd^30^. Additionally, in the *H. heidelbergensis* taxon, we included the specimens from Mauer^31^ and Kabwe^7^. The Sima de los Huesos (SH) remains have recently been removed from *Homo heidelbergensis* based on new cranial morphological and genetic data^32^. However, we include the SH specimens in the *H. heidelbergensis* taxon following the chronological context and dental morphometrics consensus regarding their classification^33^. In fact, the nuclear DNA sequences from two specimens from Sima de los Huesos show that they were related to Neandertals rather than to Denisovans^34^.

The Atapuerca–SH site is a small cavity of 8 m^2^ × 4 m^2^ that belongs to the Cueva Mayor-Cueva del Silo karst system. To date, the human fossil assemblage that has been recovered from this locality comprises approximately 6,500 remains that have been assigned to at least 28 separate individuals^27^. New U–series placed SH hominins at 434 kyr ago during MIS 12, a chronology that is consistent with the early-Middle to mid-Middle Pleistocene age for the faunal assemblage of lithostratigraphic units 6 and 7^32^. The descriptive and morphological analyses of the dental samples from the SH site until 2007 includes 533 specimens^35^.

The Arago cave site is located in the Verdouble River valley, between the towns of Tautavel and Vingrau in southern France. The human remains that have been recovered here are attributed to *H. heidelbergensis*^28^. The fauna and human fossil remains come from levels C to G. Layer C (the upper stalagmitic level, USL) corresponds to the middle Upper Mindel or the beginning of the Riss period. Layers D to G (the middle stalagmitic level, MSL) correspond to the Old Upper Mindel^36^. Layer C has an age of 120-350 kyr, Layers E and F have an age of 430±85 kyr, and Layer G (MSL) is dated to 455 kyr^37^ ago. The lower stalagmitic floor is dated between 630 ± 100 and 760 ± 80 kyr^67^ ago. The layer that yielded the human fossils is dated from 410-450 kyr^38^ ago and is placed in the Middle glacial MIS 12. The dental remains have been described in detail^29^.

The Pontnewydd human remains were recovered from a cave site that is located in the Elwy Valley, on the western edge of Vale of Clywd, approximately 6 km northwest of the town of Denbigh, England. Tooth remains come from Levels V–VI (Green, 1984). Thermoluminescence (TL) and U-series dates place the fossils in MIS 7 from 220-200 ± 25 kyr^30,39^ ago. Acheulean lithic technology corresponds to handaxes and Levallois flakes^40^.

The Mauer mandible was found in 1908 at the Rösch sand quarry in the village of Mauer, approximately 16 km southeast of Heidelberg, Germany^41^. The mandible actually presents the permanent teeth without the left premolars (P3–P4), which were lost in the 1940s^31^. The mandible has been attributed to an 18-25 year old male^42^. Paleomagnetic analyses indicated a Bruhnes polarity, which means that the mandible may be younger than 780 kyr^43^. Recently, an electron spin resonance/U-series method that is applied to mammal teeth and infrared radiofluorescence that uses sand grains dated the type-site of *H. heidelbergensis* at Mauer to 609 ± 40 kyr ago during MIS 15^31^. Paleoenvironmental conditions, according to fauna association, place the human remains in a warm and humid stage, because cold species are completely missing, and water would not have frozen permanently in winter^44^. The presence of bison and horses also indicate open landscapes that are dominated by woodlands^45^.

We placed the Kabwe (Zambia) specimen (BH1) in the *H. heidelbergensis* taxon, and this specimen is the only non-European Middle Pleistocene hominin that is included in our analyses. The human remains have been dated to approximately 200 kyr^46^ ago. The lithic materials that were found at the site are attributed to the Acheulean (Middle Stone Age)^29^.

***Homo neanderthalensis* specimens**

In our analysis, the separation between *H. neanderthalensis* (Neandertals) and *H. heidelbergensis* was based on chronological criteria, and we assigned to Neandertals the specimens that belong to the Upper Pleistocene. The Neandertal group was the only heterogeneous sample because it was built with specimens from various sites and with different chronological dates, exclusively from the Iberian Peninsula. The Neandertal group includes specimens from northern (El Sidrón) and southeastern (Sima de las Palomas and Cova Foradà) Spanish sites and from Portugal (Figueira Brava).

The El Sidrón cave (Asturias, Northern Spain) is located in a small transversal gallery (Galería del Osario) that belongs to the El Sidrón karst system. The El Sidrón site has provided a collection of Neandertal fossils of approximately 2,000 skeletal remains that represent 13 individuals who may well have died at the same time^47,48^. The age of the bone assemblage has been estimated at approximately 49 kyr (48,400 ± 3200 BP) ago during the early part of MIS^49^.

The Sima de las Palomas (SP) site is a karstic shaft in the Permo-Triassic marble of the Cabezo Gordo hill in Torre Pacheco (Murcia, Spain). Neandertal human bones and teeth have been recovered at the SP site^50,51^. A combination of accelerator mass spectrometry radiocarbon, laser ablation multicollector plasma mass spectrometry, uranium-series, optically stimulated luminescence, and a paleoclimatic correlation date the SP remains during MIS 3 with a date of less than 43,000-40,000 calendar years BP^50^.

The Cova Foradà (CF) site is a cave site that is located on the Spanish Mediterranean coast (Oliva, Valencia). The cave shows human occupation from the Mousterian to the early prehistoric and historic periods. The faunal remains from layer 11 are dated to the Upper Paleolithic period (20,119 cal BP)^52^. Human remains were discovered at level 29 and included cranial and postcranial fragments and teeth; some of these remains were isolated, and others were located in a maxilla fragment (CF-1) that has never been directly dated. Nonetheless, their morphological traits together with the lithic tools and associated faunal remains indicate a Mousterian origin for this layer^53^.

Finally, a Neandertal tooth from Portugal comes from the Figueira Brava (FB) limestone coastal cave, which is approximately 35 km southeast of Lisbon. Neandertal skeletal remains, including an upper premolar (LP^4^), were recovered from Bed 2^7,54^. The radiocarbon dates from the same layer on mollusk shells and a U-series (Th/U) on a cervid tooth place the human remains between 30.93 ± 0.7 kyr and 30.56 ± 10.7 kyr^55^ ago.

**Supplementary text 2**

**Enamel preservation and taphonomic processes**

Only well-preserved buccal enamel surfaces were studied, clearly showing *ante-mortem* microwear features and free of *post-mortem* enamel chipping, cracking, prism exposure or erosion damage. Since trampling processes can significantly alter dental enamel surfaces, discarding the teeth for microwear analysis^56,57^, special care was taken for selecting well-preserved enamel patches. *Post-mortem* processes affecting buccal enamel surfaces have been shown to be clearly distinct to *ante-mortem* diet-related microwear signatures^56–60^. Further, *in vivo* buccal microwear experimental analyses, with induced diets with distinct physical properties and abrasive particle sizes (e.g. silica phytoliths and grit), have shown that buccal enamel scratch formation increases with dietary abrasiveness^61^. This clear association between the abrasive particles content in foodstuffs and the formation of scratches on buccal enamel surfaces of living, experimental individuals (non-affected by taphonomic processes) clearly show that buccal microwear pattern formation processes are directly related to chewing of abrasive agents included with the ingested food (either intrinsic, such as phytoliths, of extrinsic as grit contaminants^61,62^). However, *post-mortem* processes, both with erosive chemicals and with abrasive particles in sediments, tend to erase or distort *ante-mortem* microwear features. Experimental analyses have already shown such distinct effect of erosive and abrasive agents on enamel surfaces^63^, as well as the association of plant material included in the calculus of human teeth and dental buccal scratches^64^. The same effects of post-depositional on enamel surfaces have been evidenced in Neandertal teeth^58^ and hominin specimens of *P. boisei* and *H. ergaster* from Laetoli and Olduvai^56^.

In the present research, a detailed analysis of the non-preserved enamel surfaces of the studied teeth was carried out (Fig. S1). Various *post-mortem* effects were readily identifiable in the samples analyzed: enamel cracks (Fig. S1a), erosion inducing enamel-prism exposure (Fig. S1b), or non-dietary related physical abrasion (Fig. S1c,d).


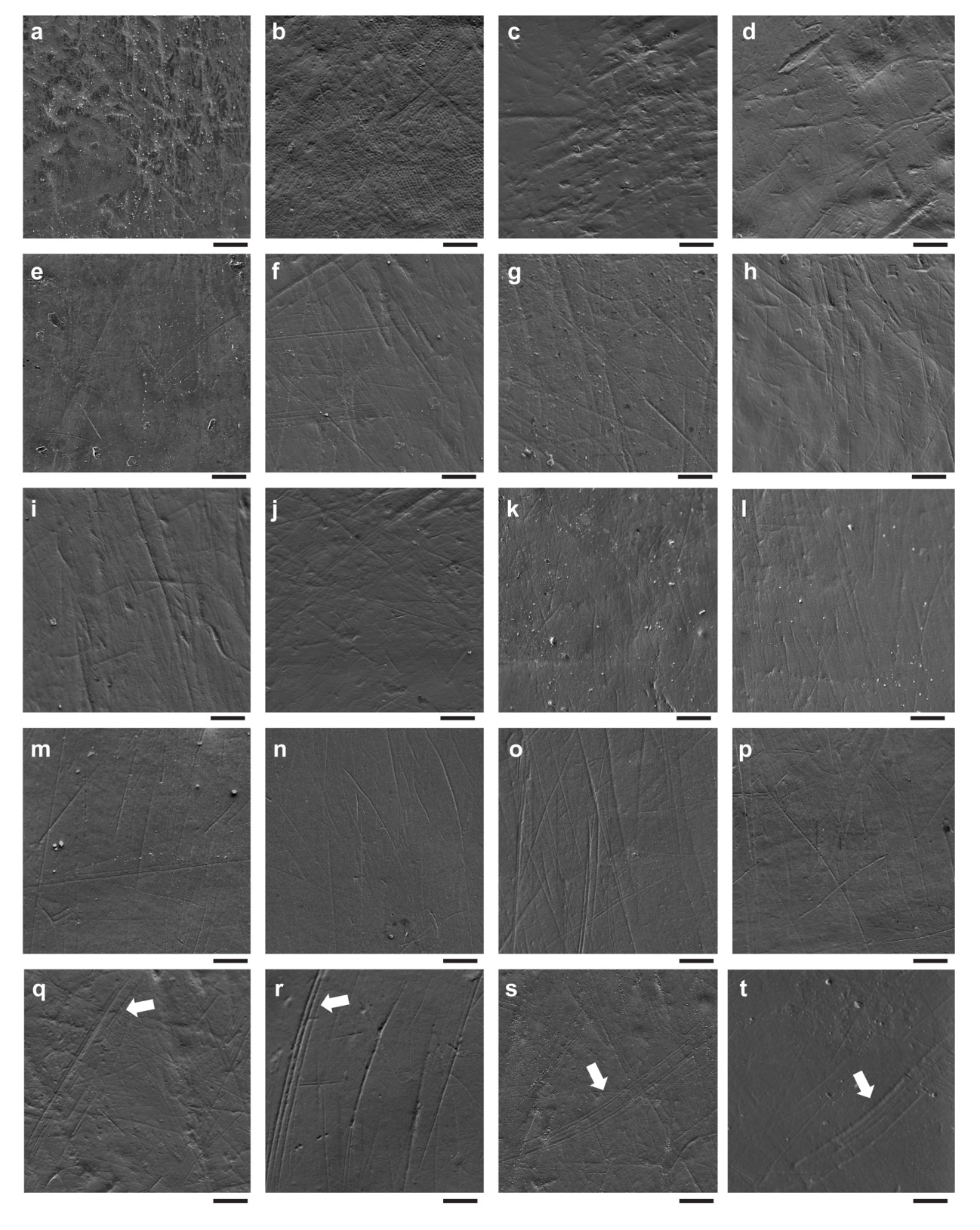


**Figure S1**. Buccal enamel surfaces showing clearly distinct microwear pattern: non-preserved enamel surfaces showing post-depositional chemical erosive and physical abrasive damages (a-d); well-preserved buccal enamel surfaces (e-p); and multi-parallel and curved scratches in both paleontological and *in vivo* human samples (q-t; see arrows). a) SP 1c (LRP4); b) ATD-6-69 (URP3); c) ATD6-113 (LLM3); d) ATD6-112 (LLm1); e) KNM-WT 15000 (LRM1); f) ATE9-1 (LLP4); g) Mauer (LRM1); h) ATD6-94 (LRM1); i) ATD6-96 (LLM1); j) ATD6-113 (LLM2); k) SDR-012 (ULM1); l) CF-1 (ULM1); m) *in vivo* 19 years old female (LLM1); n) *in vivo* 25 years old female (LLM1); o) *in vivo* 22 years old male (LLM1); p) *in vivo* 23 years old male (LLM1); q) ATD6-94 (LRM1); r) *in vivo* 25 years old female; s) ATD6-96 (LLP4); t) *in vivo* 27 years old male. Each micrograph represents an enamel patch of 0.56 mm^2^ at 100× magnification. Scale bar 100 µm (common to all images).

Similarly, well-preserved *ante-mortem* microwear patterns found could also be easily detected, showing scratches of various lengths, preferably with an occlusal to cervical orientation and a homogeneous width distribution, usually less than 20 µm^59^ (Fig. S1e-l). These well-preserved microwear patterns are similar to those recorded from *in vivo* microwear signatures in human adult volunteers of both sexes (*n* = 36; 18-35 age range) (Fig. S1m-p), used as baseline for comparative qualitative purposes^57,60^. In addition, multi-parallel and curved scratches were found in both ancient (Gran Dolina-TD6 hominins) and contemporary teeth (*in vivo* sample) (Fig. S1q-t). The similarities in their morphologies suggest that such microwear features should be attributed to *in vivo* dietary habits, as suggested for dendriform phytoliths and their *ante-mortem* scratch pattern formation capability^62,64^.

**Table S1.** Summary statistics of the buccal dental-microwear patterns for the analyzed specie or specimen.

|  |  | **Buccal dental-microwear density variables^†^** | | | | | | | | | |
| --- | --- | --- | --- | --- | --- | --- | --- | --- | --- | --- | --- |
| **Specie or specimen** | ***n*** | **NMD** | | **NV** | | **NH** | | **NDM** | | **NT** | |
|  |  | m | σ | m | σ | m | σ | m | σ | m | σ |
| *H. ergaster* | 7 | 67.14 | 22.00 | 34.71 | 22.50 | 53.86 | 23.63 | 42.71 | 29.13 | 198.43 | 81.19 |
| *ATE9-1* | 1 | 50.00 | **–** | 36.00 | **–** | 101.00 | **–** | 132.00 | **–** | 319.00 | **–** |
| *H. antecessor* | 7 | 64.29 | 35.12 | 42.43 | 18.32 | 54.57 | 24.95 | 83.57 | 40.05 | 244.86 | 83.74 |
| *Mauer* | 1 | 26.00 | **–** | 21.00 | **–** | 34.00 | **–** | 37.00 | **–** | 118.00 | **–** |
| *Sima de los Huesos* | 8 | 44.13 | 35.98 | 38.50 | 32.77 | 24.13 | 14.53 | 36.75 | 18.48 | 143.50 | 57.34 |
| *Arago* | 6 | 29.33 | 18.58 | 25.83 | 23.80 | 30.83 | 19.55 | 46.33 | 32.71 | 132.33 | 71.14 |
| *Pontnewydd* | 5 | 62.40 | 45.75 | 51.20 | 48.13 | 46.00 | 23.05 | 34.40 | 27.23 | 194.00 | 85.16 |
| *BH1* | 1 | 16.00 | **–** | 15.00 | **–** | 60 | **–** | 65 | **–** | 156.00 | **–** |
| *Neanderthal* | 6 | 52.33 | 30.85 | 35.33 | 30.19 | 33.67 | 39.58 | 20.17 | 12.61 | 141.50 | 75.61 |
|  |  | **Buccal dental-microwear length variables (in μm)^†^** | | | | | | | | | |
| **Specie or specimen** | ***n*** | **XMD** | | **XV** | | **XH** | | **XDM** | | **XT** | |
|  |  | m | σ | m | σ | m | σ | m | σ | m | σ |
| *H. ergaster* | 7 | 70.98 | 18.56 | 105.41 | 60.07 | 78.95 | 15.23 | 64.76 | 21.03 | 79.23 | 21.36 |
| *ATE9-1* | 1 | 88.30 | **–** | 92.92 | **–** | 113.08 | **–** | 65.76 | **–** | 87.34 | **–** |
| *H. antecessor* | 7 | 92.48 | 28.59 | 123.84 | 28.95 | 107.57 | 21.82 | 83.62 | 26.39 | 94.37 | 17.96 |
| *Mauer* | 1 | 136.34 | **–** | 203.06 | **–** | 125.59 | **–** | 157.22 | **–** | 151.67 | **–** |
| *Sima de los Huesos* | 8 | 67.58 | 16.53 | 88.51 | 31.73 | 100.73 | 38.69 | 87.68 | 15.75 | 88.41 | 12.69 |
| *Arago* | 6 | 93.65 | 25.99 | 118.86 | 61.47 | 116.68 | 48.04 | 73.34 | 21.38 | 100.96 | 31.73 |
| *Pontnewydd* | 5 | 93.00 | 35.20 | 94.67 | 20.03 | 95.89 | 11.63 | 94.60 | 15.59 | 91.29 | 14.47 |
| *BH1* | 1 | 72.64 | **–** | 94.68 | **–** | 90.10 | **–** | 96.62 | **–** | 91.47 | **–** |
| *Neanderthal* | 6 | 122.69 | 102.73 | 128.62 | 40.37 | 104.36 | 30.81 | 69.59 | 30.74 | 100.43 | 28.99 |
|  |  | **Standard deviation of microwear length variables^†^** | | | | | | | | | |
| **Specie or specimen** | ***n*** | **SMD** | | **SV** | | **SH** | | **SDM** | | **ST** | |
|  |  | m | σ | m | σ | m | σ | m | σ | m | σ |
| *H. ergaster* | 7 | 77.71 | 34.14 | 101.62 | 67.15 | 70,05 | 16.18 | 73.4 | 50.54 | 87.85 | 31.45 |
| *ATE9-1* | 1 | 75.12 | – | 107.7 | **–** | 117,37 | **–** | 66.31 | **–** | 93.26 | **–** |
| *H. antecessor* | 7 | 100.38 | 26.19 | 120.9 | 25.89 | 128,03 | 31.36 | 84.45 | 32.01 | 108.11 | 17.34 |
| *Mauer* | 1 | 130.92 | – | 191.64 | – | 136.16 | – | 172.21 | – | 158.20 | – |
| *Sima de los Huesos* | 8 | 68.93 | 31.31 | 77.82 | 37.89 | 102.84 | 57.76 | 85.10 | 29.37 | 92.93 | 17.96 |
| *Arago* | 6 | 96.33 | 50.92 | 125.36 | 69.15 | 121.56 | 65.52 | 72.96 | 29.34 | 109.34 | 46.09 |
| *Pontnewydd* | 5 | 106.87 | 59.16 | 87.81 | 35.98 | 97.85 | 23.74 | 94.45 | 14.17 | 97.69 | 18.44 |
| *BH1* | 1 | 60.45 | – | 75.40 | – | 80.41 | – | 160.76 | – | 118.53 | – |
| *Neanderthal* | 6 | 112.75 | 53.32 | 125.21 | 47.61 | 115,74 | 58.45 | 75.77 | 48.45 | 116.27 | 30.05 |

^†^ The data show the mean (m) and standard deviation (σ) of the number (N) and length (X; in μm) of the enamel scratches that are classified into four orientation categories (V, M, D and H) of 45º intervals, and all categories are pooled (T). A total of 15 variables of scratch density (NM, NV, NH, ND and NT), length (XM, XV, XH, XD and XT) and length standard deviation (SM, SV, SH, SD and ST) were derived for the studied sample.

**Table S2.** (see suppl. Excel file).

**Table S3. Interspecific statistical comparisons for all of the considered buccal microwear variables.**

| **MANOVA** | **Value** | **df** | **F** | ***P*** |
| --- | --- | --- | --- | --- |
| Wilks’ *λ* | 0.108 | 42, 74 | 1.994 | 0.005 |
| Pillai trace | 1.496 | 42, 81 | 1.917 | 0.006 |
| Hotelling–Lawley | 3.593 | 42, 71 | 2.025 | 0.004 |
|  |  |  |  |  |
| **ANOVAs** |  |  |  |  |
| **Variable** | **Mean Square** | **df** | **F** | ***P*** |
| NH | 1,881.6 | 3, 38 | 2.913 | **0.047** |
| XH | 1,399.51 | 3, 38 | 1.583 | 0.210 |
| SH | 4,362.59 | 3, 38 | 2.207 | 0.103 |
| NV | 81.51 | 3, 38 | 0.098 | 0.961 |
| XV | 1,214.13 | 3, 38 | 0.601 | 0.618 |
| SV | 1,569.56 | 3, 38 | 0.601 | 0.618 |
| NMD | 1,520.33 | 3, 38 | 1.504 | 0.229 |
| XMD | 3,186.4 | 3, 38 | 1.594 | 0.207 |
| SMD | 1,503.52 | 3, 38 | 0.848 | 0.477 |
| NDM | 6,642.88 | 3, 38 | 8.592 | **0.000** |
| XDM | 1,319.56 | 3, 38 | 2.163 | 0.108 |
| SDM | 808.13 | 3, 38 | 0.526 | 0.667 |
| NT | 23,925.34 | 3, 38 | 4.430 | **0.009** |
| XT | 608.9 | 3, 38 | 1.171 | 0.333 |
| ST | 915.94 | 3, 38 | 1.117 | 0.354 |

df: degrees of freedom, F: ANOVA statistic, *P*: significance probability at *P* < 0.05 (in bold)

**Table S4.** Results (the Eigenvalues and Pearson coefficients of correlation) of the Canonical Variates Analysis (CVA) on the buccal dental-microwear patterns for the considered groups.

|  | **CV1** | **CV2** | **CV3** |
| --- | --- | --- | --- |
| Eigenvalue | 2.387 | 1.498 | 1.006 |
| % Explained variance | 43.153 | 27.094 | 18.192 |
| % Cumulative variance | 43.153 | 70.247 | 88.438 |
|  |  |  |  |
| **Variable** | ***r*** | ***r*** | ***r*** |
| NH | −0.172 | 0.457** | 0.165 |
| XH | 0.202 | −0.027 | −0.311 |
| SH | 0.185 | 0.103 | −0.236 |
| NV | 0.054 | 0.035 | 0.312 |
| XV | −0.192 | 0.223 | −0.271 |
| SV | −0.187 | 0.215 | −0.368* |
| NMD | −0.183 | 0.295 | 0.395* |
| XMD | −0.236 | −0.011 | −0.139 |
| SMD | −0.177 | 0.047 | −0.081 |
| NDM | 0.330* | 0.680** | −0.090 |
| XDM | 0.392* | −0.099 | 0.319* |
| SDM | 0.132 | −0.026 | 0.214 |
| NT | 0.024 | 0.553** | 0.286 |
| XT | 0.025 | −0.063 | −0.266 |
| ST | −0.076 | 0.051 | −0.244 |

CVA included six groups and 15 buccal microwear density and length (in μm) variables (see Table 1 and Methods for the groups and variable description details, respectively). This table displays the first three canonical variates (CV) that were obtained, their eigenvalues, and the percentage of the explained variance. The original CVA extracted five variables that explain the total variation of the analyzed subsample. The CV4 (7.60%) and CV5 (3.95%) explained less than 10% of the variance. There were significant Pearson correlation (*r*) values of *P* < 0.05* and *P* < 0.01**.

**Table S5.** Original and cross-validated percentages (%) of correct assignment obtained from the Canonical Variates Analysis (CVA).

| **Specie** | **Original (%)** | **Nº of correctly assigned specimens** | **Cross-validated (%)** | **Nº of correctly assigned specimens** |
| --- | --- | --- | --- | --- |
| *H. ergaster* | 85.71 | 6/7 | 57.14 | 4/7 |
| *H. antecessor* | 85.71 | 6/7 | 57.14 | 4/7 |
| Arago | 66.67 | 4/6 | 16.67 | 1/6 |
| Sima de los Huesos | 87.50 | 7/8 | 50.00 | 4/8 |
| Pontnewydd | 40.00 | 2/5 | 0.00 | 0/5 |
| Iberian Neandertals | 66.67 | 4/6 | 0.00 | 0/6 |
| Total | 74.36 |  | 33.33 |  |

The isolated specimen ATE9−1 (*H. sp*) was classified as *H. antecessor* (0.88%), and the assigned *H. heidelbergensis* Kabwe (BH1) and Mauer (Ma) specimens were classified as *H. ergaster* (0.95%) and Sima de los Huesos (0.44%), respectively.

**Supplementary Information References**

1. Antón, S. C. Natural history of *Homo erectus. Yrbk. Phys. Anthropologist* **46**, 126–170 (2003).

2. Antón, S. C., Potts, R. & Aiello, L. C. Human evolution. Evolution of early *Homo*: an integrated biological perspective. *Science* **345**, 1236828 (2014).

3. Wood, B. *Koobi Fora Research Project, vol. 4: Hominid Cranial Remains* (Clarendon Press Oxford, 1991).

4. Brown, F. H. & McDougall, I. *The Nariokotome Homo Erectus Skeleton: Geological Setting and Age* (ed Walker, A., & Leakey, R.) 9–20 (Harvard University Press Cambridge, 1993).

5. Cerling, T. E. *et al.* Stable isotope-based diet reconstructions of Turkana basin hominins. *Proc. Natl. Acad. Sci. U.S.A.* **110**, 10501–10506 (2013).

6. Leakey, M. D. & *Gorge, O. Excavations in Beds I And II, 1960–1963* (Cambridge University Press, 1971).

7. Schwartz, J. H. & Tattersall, I. *The Human Fossil Record, Craniodental Morphology of Genus* Homo *(Africa and Asia)* *Vol 2.* (Wiley, 2003).

8. Grine, F. E. Early Homo at Swartkrans, South Africa: a review of the evidence and an evaluation of recently proposed morphs. *South. Afr. J. Sci.* **101**, 43–52 (2005).

9. Ungar, P. S. Dental evidence for the reconstruction of diet in African early *Homo. Curr. Anthropol.* **53**, 318–329 (2012).

10. Bermúdez de Castro, J. M. *et al.* Early Pleistocene human mandible from Sima del Elefante (TE) cave site in Sierra de Atapuerca (Spain): A comparative morphological study*. J. Hum. Evol.* **61**, 12–25 (2011).

11. Carbonell, E. *et al.* The first hominin of Europe. *Nature* **452**, 465–469 (2008).

12. Bermúdez de Castro, J. M. *et al.* A hominid from the lower Pleistocene of Atapuerca: possible ancestor to neandertals and modern humans. *Science* **276**, 1392–1395 (1997).

13. Huguet, R. *et al.* Successful subsistence strategies of the first humans in south-western Europe. *Quat. Int.* **295**, 168–182 (2013).

14. De Lombera-Hermida, A. *et al.* The lithic industry of Sima del Elefante (Atapuerca, Burgos, Spain) in the context of early and middle Pleistocene technology in Europe.  *J. Hum. Evol.* **82**, 95–106 (2015).

15. Parés, J. M. & Pérez-González, A. Magnetochronology and stratigraphy at Gran Dolina section, Atapuerca (Burgos, Spain). *J. Hum. Evol.* **37**, 325–342 (1999).

16. Carbonell, E. *et al.* The TD6 level lithic industry from Gran Dolina, Atapuerca (Burgos, Spain): production and use. *J. Hum. Evol.* **37**, 653–693 (1999).

17. García, N. & Arsuaga, J. L. Les carnivores (Mammalia) des sites du Pleistocene ancien et moyen d'Atapuerca (Espagne). *L'Anthropologie* **105**, 83–93 (2001).

18. Van der Made, J. Les ongulés d’Atapuerca. Stratigraphie et biogéographie. *L'Anthropologie* **105**, 95–113 (2001).

19. Bermúdez De Castro, J. M., Rosas, A. & Nicolás, M. E. Dental remains from Atapuerca-TD6 (Gran Dolina site, Burgos, Spain). *J. Hum. Evol.* **37**, 523–566 (1999).

20. Bermúdez de Castro, J. M. *et al.* A new early Pleistocene hominin mandible from Atapuerca-TD6, Spain. *J. Hum. Evol.* **55**, 729–735 (2008).

21. Bermúdez de Castro, J. M. *et al.* New immature hominin fossil from European lower Pleistocene shows the earliest evidence of a modern human dental development pattern. *Proc. Natl Acad. Sci. USA* **107**, 11739–11744 (2010).

22. Carbonell, E. *et al.* An early Pleistocene hominin mandible from Atapuerca-TD6, Spain. *Proc. Natl Acad. Sci. USA* **102**, 5674–5678 (2005).

23. Gómez-Robles, A., Bermúdez De Castro, J. M., Martinón-Torres, M. & Prado-Simón, L. Crown size and cusp proportions in *Homo antecessor* upper first molars. A comment on Quam *et al*. *J. Anat.* **218**, 258–262 (2011).

24. Moreno, D. *et al.* New radiometric dates on the lowest stratigraphical section (TD1 to TD6) of Gran Dolina site (Atapuerca, Spain). *Quat.* *Geochronol.* **30** Part B, 535–540 (2015).

25. Parés, J. M. *et al.* Reassessing the age of Atapuerca-TD6 (Spain): New paleomagnetic results. *J. Archaeol. Sci.* **40**, 4586–4595 (2013).

26. Carbonell, E. *et al.* Cultural cannibalism as a Paleoeconomic system in the European lower Pleistocene. *Curr. Anthropol.* **51**, 539–549 (2010).

27. Bermúdez de Castro, J. M., Martinón-Torres, M., Lozano, M., Sarmiento, S. & Muela, A. Paleodemography of the Atapuerca-Sima de los Huesos hominin sample: a revision and new approaches to the paleodemography of the European middle Pleistocene population. *J. Anthropol. Res.* **60**, 5-26 (2004).

28. Tattersall, I. *The Fossil Trail: How We Know What We Think We Know about Human Evolution* (Oxford University Press, 1995).

29. Schwartz, J. H. & Tattersall, I. *The Human Fossil Record, Terminology and Craniodental Morphology of Genus* Homo *(Europe) Vol 1* (Wiley, 2002).

30. Green, H. S. *et al.* Pontnewydd Cave in Wales—a new middle Pleistocene hominid site. *Nature* **294**, 707–713 (1981).

31. Wagner, G. A. *et al.* Radiometric dating of the type-site for *Homo heidelbergensis* at Mauer, Germany. *Proc. Natl Acad. Sci. USA.* **107**, 19726–11930 (2010).

32. Arsuaga, J. L. *et al.* Neandertal roots: cranial and chronological evidence from Sima de los Huesos. *Science* **344**, 1358–1363 (2014).

33. Gómez-Robles, A., Bermúdez de Castro, J. M., Martinón-Torres, M., Prado-Simón, L. & Arsuaga, J. L. A geometric morphometric analysis of hominin lower molars: evolutionary implications and overview of postcanine dental variation. *J. Hum. Evol.* **82**, 34–50 (2015).

34. Meyer, M. *et al.* Nuclear DNA sequences from the middle Pleistocene Sima de los Huesos hominins. *Nature* **531**, 504–507 (2016).

35. Martinón-Torres, M., Bermúdez de Castro, J. M., Gómez-Robles, A., Prado-Simón, L. & Arsuaga, J. L. Morphological description and comparison of the dental remains from Atapuerca-Sima de los Huesos site (Spain). *J. Hum. Evol.* **62**, 7–58 (2012).

36. De Lumley, M. A. & de Lumley, H. Découverte de restes humains anténéandertaliens du début du Riss à la Caune de l'Arago. *C. R. Acad. Sci.* **272**, 1739–1742 (1971).

37. Yokoyama, Y., Falguères, C. & Quaegebeur, J. P. ESR dating of quartz from quaternary sediments: first attempt. *Nucl. Tracks* **10**, 921–928 (1985).

38. Iacumin, P., Cominotto, D. & Longinelli, A. A stable isotope study of mammal skeletal remains of mid-Pleistocene age, Arago cave, eastern Pyrenees, France. Evidence of taphonomic and diagenetic effects. *Palaeogeogr. Palaeoclimatol. Palaeoecol.* **126**, 151–160 (1996).

39. Aldhouse–Green, S., Pettitt, P. & Stringer, C. Holocene humans at Pontnewydd and Cae Gronw caves. *Antiquity* **70**, 444–447 (1996).

40. Aldhouse–Green, S. H. R. *Human Evolution in Europe and the Atapuerca Evidence.* *Vol.* *1: Pontnewydd Cave, Wales; a Later Middle Pleistocene Hominid and Archeological Site: a Review of Stratigraphy, Dating, Taphonomy and Interpretation* (eds Bermúdez de Castro, J. .M, Arsuaga, J.L., Carbonell, E.) 37–55 (Junta de Castilla y León, Consejería de Cultura y Turismo, Valladolid, 1995).

41. Schoetensack, O. *The Mandible of Homo Heidelbergensis from the Sands of Mauer near Heidelberg. A Contribution to the Palaeontology of Man* (Engelmann and Leipzig, 1908).

42. Condemi, S. & von Koenigswald, W. *Homo heidelbergensis von Mauer: Der Unterkiefer von Mauer* (eds Wagner, G. A., & Beinhauer, K. W.) 200–214 (Universitätsverlag, 1997).

43. Hambach, U. Paläo- und gesteinsmagnetische Untersuchungen im Quartärder Grube Grafenrain: Fundplatz des *Homo erectus heidelbergensis*. *MannheimerGeschichtsblätter Beiheft* **1**, 41–46 (1996).

44. Kahlke, R.-D. *et al.* Western Palaearctic paleoenvironmental conditions during the Early and early Middle Pleistocene inferred from large mammal communities, and implications for hominin dispersal in Europe. *Quat. Sci. Rev.* **30**, 1368–1395 (2011).

45. Wagner, G. A., Maul, L. C., Löscher, M. & Schreiber, H. D. Mauer, the type site of *Homo heidelbergensis*: Palaeoenvironment and age. *Quat. Sci. Rev.* **30**(11-12), 1464–1473 (2010).

46. Henke, W. *Handbook of Paleoanthropology; Historical Overview of Paleoanthropologucal Research* (eds Henke, W., & Tattersall, I.) 1–56 (Springer, 2007).

47. Lalueza-Fox, C., Rosas, A. & de la Rasilla, M. Palaeogenetic research at the El Sidrón Neanderthal site. *Ann. Anat.* **194**, 133–137. 2012.

48. Rosas, A. *et al.* Les Néandertaliens d’El Sidrón (Asturies, Espagne). Actualisation d’un nouvel échantillon. *L’Anthropologie* **116**, 57–76 (2012).

49. Wood, R. E. *et al.* A new date for the neanderthals from El Sidrón cave (Asturias, Northern Spain). *Archaeometry* **55**, 148–158 (2013).

50. Walker, M. J. *et al.* Late Neandertals in southeastern Iberia: Sima de las Palomas del Cabezo Gordo, Murcia, Spain. *Proc Natl Acad Sci USA* **105**, 20631–20636 (2008).

51. Walker, M. J., Ortega, J., Parmová, K., López, M. V. & Trinkaus, E. Morphology, body proportions, and postcranial hypertrophy of a female Neandertal from the Sima de las Palomas, southeastern Spain. *Proc. Natl. Acad Sci. USA* **108**, 10087–1091 (2011).

52. Aparicio, J. *et al. Los Neandertales de la Cova Foradà de Oliva*. 73 pp. (Real Academia de Cultura Valenciana, 2014).

53. Lozano, M., Subirà, M. E., Aparicio, J., Lorenzo, C. & Gómez-Merino, G. Toothpicking and periodontal disease in a Neanderthal specimen from Cova Foradà site (Valencia, Spain). *PLoS ONE* **8**, e76852 (2013).

54. Antunes, M. *The Pleistocene Fauna From Gruta do Figueira Brava: a Synthesis. Last Neanderthals in Portugal: Odontologic and Other Evidence* (ed Antunes, M.) 259–282 (Memórias da Academia das Ciências de Lisboa, 2000).

55. Antunes, M., Cunha, A. Neanderthalian remains from Figueira Brava Cave, Portugal. *Geobios* **25**, 681–692 (1992).

56. Martínez, L. M. & Pérez-Pérez, A. *Post-mortem* wear as indicator of taphonomic processes affecting enamel surfaces of hominin teeth from Laetoli and Olduvai (Tanzania): implications to dietary interpretations. *Anthropologie* **42**, 37–42 (2004).

57. Romero, A. &, De Juan, J. *Scanning Electron Microscopy for the Life Sciences: SEM, Teeth and Palaeoanthropology: the Secret of Ancient Human Diets* (ed Schatten, H.) 236–256 (Cambridge University Press, 2012).

58. Pérez-Pérez, A., Espurz, V., Bermúdez de Castro, J. M., de Lumley, M. A. & Turbón, D. Non-occlusal dental microwear variability in a sample of middle and late Pleistocene human populations from Europe and the near east. *J. Hum. Evol.* **44**, 497–513 (2003).

59. Lalueza Fox, C. & Pérez-Pérez, A. Cutmarks and p*ost-mortem* striations in fossil human teeth. *Hum. Evol.* **9**, 165–172 (1994).

60. Romero, A. & De Juan, J. Intra- and interpopulation human buccal tooth surface microwear analysis: inferences about diet and formation processes. *Anthropologie* **45**, 61–70 (2007).

61. Romero, A., Galbany, J., De Juan, J. & Pérez-Pérez, A. Short and long-term *in* *vivo* human buccal dental-microwear turnover. *Am. J. Phys. Anthropol.* **148**, 467–472 (2012).

62. Lalueza, C., Pérez-Pérez, A. & Juan J. 1994. Dietary information through the examination of plant phytoliths on the enamel surface of human dentition. *J. Archaeol. Sci.* **21**, 29–34 (1994).

63. King, T., Andrews, P. & Boz, B. Effect of taphonomic processes on dental microwear. *Am. J. Phys. Anthropol.* **108**, 359–373 (1999).

64. Buchet, L., Cremoni, N., Rucker, C. & Verdin, P. *Phytoliths - Applications in Earth Science and Human History: Comparison between the distribution of dental micro-striations and plant material included in the calculus of human teeth* (ed Colin, F.) 107–117 (Taylor & Francis, 2001).
